# Supplementary material for: Epidemiology of Severe Fever with Thrombocytopenia Syndrome in Dogs and Cats in Taiwan
Source: Viruses. 2023 Nov 28;15(12):2338. doi: 10.3390/v15122338 (PMC10747826; doi:10.3390/v15122338)
Supplement: Supplementary file 1 [file viruses-15-02338-s001.zip › viruses-2718535-supplementary.pdf]

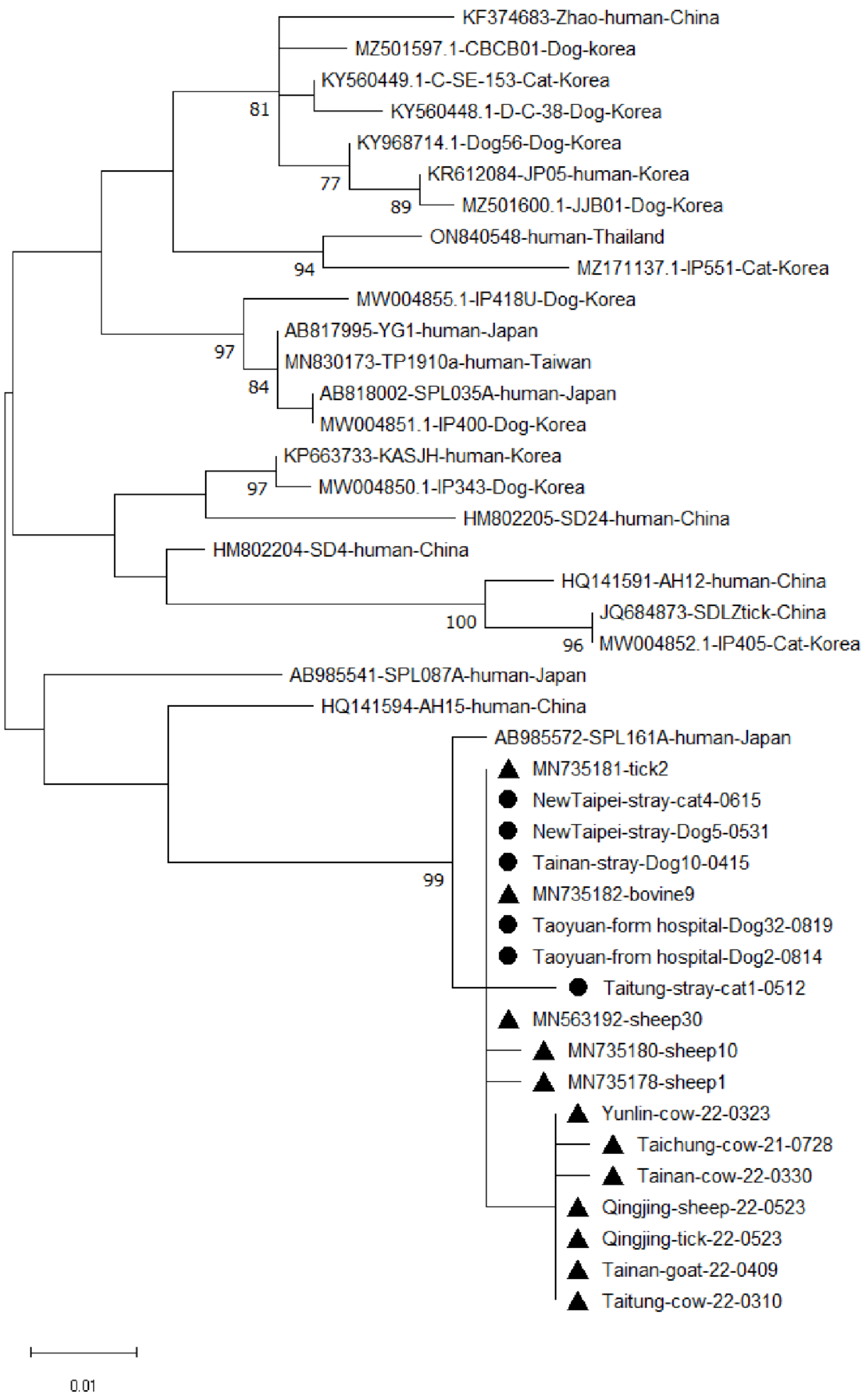

Supplementary Figure S1. Phylogenetic analysis of the partial NP of SFTSV identified in dogs and cats in Taiwan. In total, the sequences from six samples with higher RNA loads obtained from dogs and cats (indicated by a black circle) were further analyzed. Notably, these strains were clustered together with those amplified from ruminants and ticks in Taiwan (indicated by a triangle). Representative viral strains, along with their accession numbers and the host and country of isolation, were included. The phylogenetic analysis utilized the maximum-likelihood method, based on the Kimura 2-parameter model, with 1000 bootstrap replicates; bootstraps higher than 75 were shown. The percentage of trees in which associated taxa clustered is indicated next to the branches, and the scale bar represents nucleotide substitutions per position.
